# Implant-Derived *S. aureus* Isolates Drive Strain-Specific Invasion Dynamics and Bioenergetic Alterations in Osteoblasts

**DOI:** 10.3390/antibiotics14020119

**Published:** 2025-01-23

**Authors:** Lei Song, Lea-Sophie Schwinn, Juliane Barthel, Vanessa Ketter, Philipp Lechler, Uwe Linne, Ardawan J. Rastan, Sebastian Vogt, Steffen Ruchholtz, Jürgen R. J. Paletta, Madeline Günther

**Affiliations:** 1 Center of Orthopedics and Trauma Surgery, Philipps-University Marburg, Universitätsklinikum Gießen and Marburg GmbH, 35043 Marburg, Germany; 2 Faculty of Chemistry, Philipps-University Marburg, 35032 Marburg, Germany; 3 Department of Cardiac and Thoracic Vascular Surgery, Philipps-University Marburg, Universitätsklinikum Gießen and Marburg GmbH, 35043 Marburg, Germany

**Keywords:** implant infections, *S. aureus*, clinical isolates, SaOS2 and MG63 osteoblasts, adhesion-, invasion- and survival assay, mitochondrial respiration

## Abstract

**Background:** Implants are integral to modern orthopedic surgery. The outcomes are good, but infections remain a serious issue. *Staphylococcus aureus* (*S. aureus*), along with *Staphylococcus epidermidis*, are predominant pathogens responsible for implant-associated infections, as conventional antibiotic treatments often fail due to biofilm formation or the pathogens’ ability to invade cells and to persist intracellularly. **Objectives:** This study therefore focused on interactions of *S. aureus* isolates from infected implants with MG63 and SaOS2 osteoblasts by investigating the adhesion, invasion, and the impact on the bioenergetics of osteoblasts. **Methods and Results:** We found that the ability of *S. aureus* to adhere to osteoblasts depends on the isolate and was not associated with a single gene or expression pattern of characteristic adhesion proteins, and further, was not correlated with invasion. However, analysis of invasion capabilities identified better invasion conditions for *S. aureus* isolates with the SaOS2 osteoblastic cells. Interestingly, metabolic activity of osteoblasts remained unaffected by *S. aureus* infection, indicating cell survival. In contrast, respiration assays revealed an altered mitochondrial bioenergetic turnover in infected cells. While basal as well as maximal respiration in MG63 osteoblasts were not influenced statistically by *S. aureus* infections, we found increased non-mitochondrial respiration and enhanced glycolytic activity in the osteoblasts, which was again, more pronounced in the SaOS2 osteoblastic cells. **Conclusions:** Our findings highlight the complexity of *S. aureus*-host interactions, where both the pathogen and the host cell contribute to intracellular persistence and survival, representing a major factor for therapeutic failures.

## 1. Introduction

The growing prevalence of endoprosthesis implantations, driven by aging populations and increasing activity levels among the elderly, has led to a significant rise in surgical procedures globally. Total hip and knee replacements alone account for approximately 1,000,000 and 250,000 procedures annually worldwide [1]. In the United States, the number of total joint arthroplasties performed each year exceeds one million [2], and projections suggest a substantial increase to nearly four million procedures by 2030 [3]. Prosthetic implants typically have an average lifespan of 15 years, with aseptic loosening being the primary cause of failure [4,5]. In addition, implant-associated infections are becoming increasingly important [6,7].

These infections often lead to enhanced morbidity, delayed healing, and impaired nonunion of fractures, frequently necessitating aggressive interventions such as debridement, revision surgeries, and prolonged antimicrobial therapy [8]. Currently, the emergence of antimicrobial-resistant strains complicates treatment strategies, often resulting in chronic or relapsing infections [1,9].

Among the various pathogens implicated in implant-associated infections, *S. aureus* has emerged as a leading cause. Known for its virulence and robust pathogenicity, *S. aureus* plays a central role in orthopedic infections [10,11,12,13,14]. While *S. aureus* commonly colonizes human skin and mucous membranes, with approximately 20% of the population being persistent bacterial strains, it can transition from a commensal organism to a pathogen under certain conditions [15], leading to a wide range of infections from superficial skin lesions to life-threatening diseases such as endocarditis and sepsis [16].

A key mechanism by which *S. aureus* establishes and sustains implant-associated infections is biofilm formation [17]. Biofilms, composed of bacterial aggregates adhering to the prosthetic surface, form a resilient matrix that protects the bacteria from host defenses and antimicrobial agents, making eradication exceedingly difficult [18,19]. The biofilm lifecycle includes initial adhesion, micro-colony formation, maturation, and bacterial dispersal to distant sites [20,21,22].

In addition to biofilm formation, *S. aureus* has the ability to invade non-professional phagocytes, such as osteoblasts [23,24,25,26,27]. This intracellular invasion allows *S. aureus* to evade host immune responses and resist anti-microbial therapies, which have profound effects on osteoblast function and bone health [10,28,29,30,31]. This ability to survive intracellularly is a key factor in the persistence of orthopedic implant infections, highlighting the need to target intracellular bacteria to prevent recurrence [32,33,34,35,36].

Given these challenges, the primary objective of this study was to characterize clinical isolates of *S. aureus* associated with implant-related infections, with a focus on their adhesion and invasion into osteoblasts. Special attention was given to mitochondrial respiration as a critical factor in the infection process. To achieve this, we used an established infection model to investigate the invasion and persistence of *S. aureus* within MG63 and SaOS2 osteoblasts and assessed mitochondrial respiration and glycolytic activity in these cells infected with implant-derived *S. aureus* isolates.

## 2. Results

### 2.1. Charakterisation of Patient-Derived S. aureus Isolates Used for Infection of Osteoblasts

**Figure 1:**
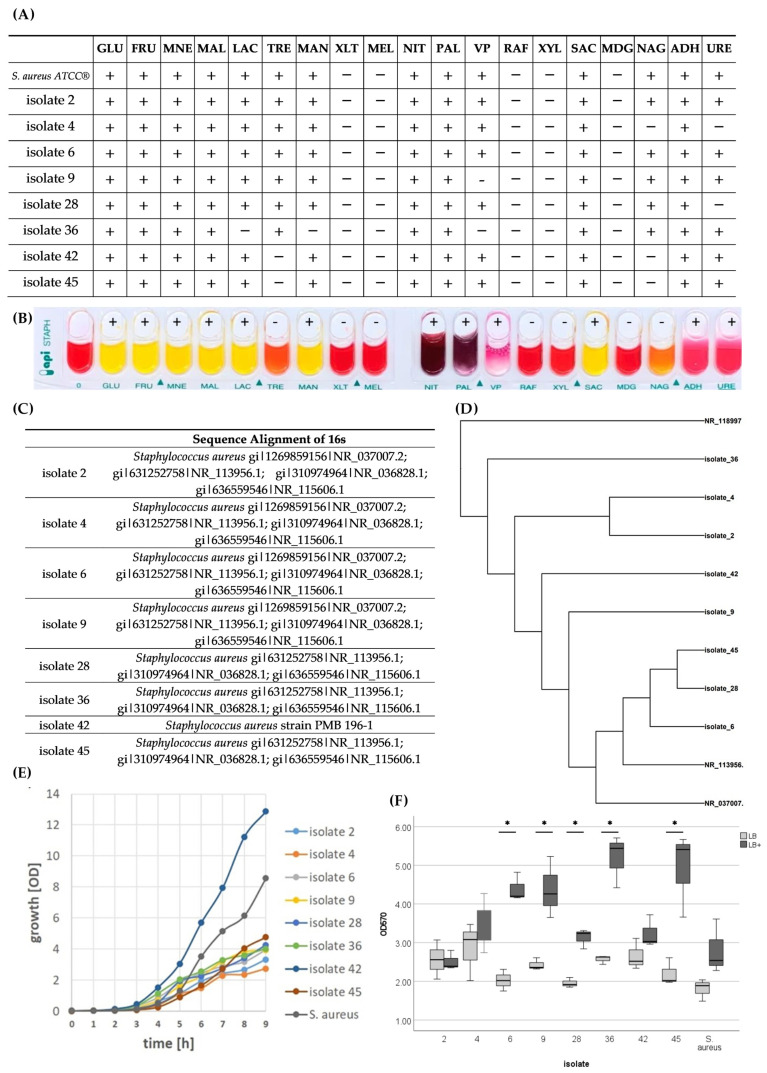
Characterization of *S. aureus* isolates from implant associated infections. (**A**) Result of the API^®^ Staph. test of isolates obtained from patients that underwent surgery for an implant-associated infection. (**B**) Color-changing strip of patient 42 is presented as an example. (**C**) Homologies of *S. aureus* isolates based on 16S rRNA sequencing, and (**D**) phylogenetic tree based on DNA likelihood with molecular clock. (**E**) Growth curve of the isolates in LB medium. (**F**) Biofilm formation of the isolates in LB and LB medium supplemented with NaCl and glucose. 0: blank; GLU: D-Glucose; FRU: D-Fructose; MNE: D-Mannose; MAL: D-Maltose; LAC: D-Lactose; TRE: D-Trehalose; MAN: D-Mannitol; XLT: Xylitol; MEL: D-Melibiose: NIT: Kaliumnitrat: PAL: ß-Naphthol-Phosphat; VP: Voges Proskauer; RAF: D-Raffinose; XYL: D-Xylose; SAC: D-Saccharose; MDG: Methyl-αD Glucopyranoside; NAG: N-acetyl-glucosamine; ADH: Arginine DiHydrolase; URE: UREase; +: Positive; −: Negative.

*S. aureus* strains were isolated from patients during 2017–2018, obtained from four men and four women with a mean age of 67.1 years, all of whom underwent revision surgery due to implant-associated infection. All isolates were collagenase positive and showed similar but not identical metabolic activity with respect to substrate utilization (Figure 1A,B) and related to 16S rRNA (Figure 1C,D), indicating the presence of different subtypes of *S. aureus*.

With regard to bacterial growth, all isolates, with the exception of isolate 42, showed slower proliferation rates than the laboratory strain (Figure 1E). With regard to biofilm formation, no difference was found in the basal formation after one day of cultivation. However, biofilm formation could be induced by the composition of the growth medium in some (6, 9, 28, 36, and 45) but not all (2, 4, and 42) isolates (Figure 1F).

### 2.2. Patient-Derived S. aureus Isolates Showed Heterogenous Adhesion- and Invasion Properties in the SaOS2 and MG63 Osteoblasts

**Figure 2:**
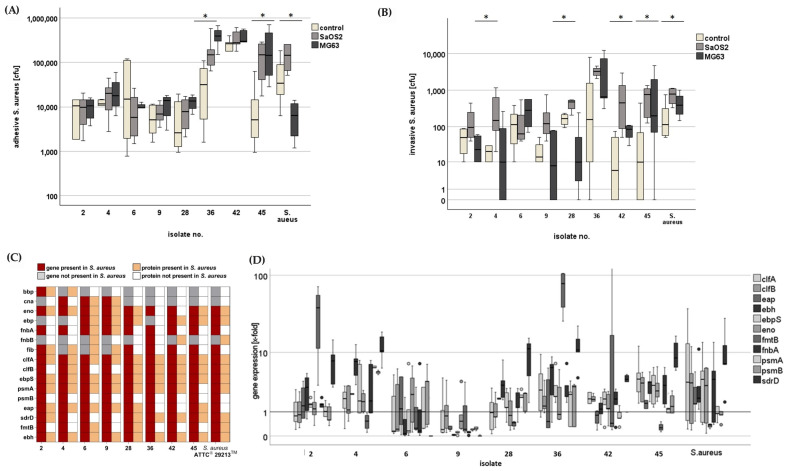
Adhesion and invasion of *S. aureus* isolates on osteoblast like cell lines. (**A**) SaOS2 and MG63 were grown to confluence in 6-well plates and then incubated with *S. aureus* isolates from stationary growth phase. After 30 min, non-adherend bacteria were washed away with PBS. Then, osteoblasts were lysed and released bacteria were plated on LB-agar in order to determine colony-forming units. * *p* < 0.05 compared to plastic surface (control). (**B**) Invasion assay of *S. aureus* isolates in SaOS2 and MG63 cell lines. After adhesion and washing, cells were incubated in DMEM supplemented with 10% FCS and 30 μg/mL gentamicin overnight in order to selectively eliminate remaining extracellular bacteria by quantifying colony-forming units. * *p* < 0.05 compared to plastic surface (control). (**C**) Genes and proteins involved in the adhesion of *S. aureus* isolates as determined by PCR or mass spectrometry. (**D**) Genes for adhesion molecules found in *S. aureus* isolates were analyzed with respect to their expression level using qPCR; bbp encoding bone sialoprotein binding protein, cna collagen binding protein, eno laminin binding protein, fnbA and fnbB fibronectin binding proteins A and B, fib fibrinogen binding protein, clfA and clfB clumping factors A and B, ebpS elastin binding protein; psmA and psmB Phenol-soluble modulins A and B; eap extracellular adher-ence protein; sdrD SD-repeat containing protein D; fmtB methicillin resistance determinant FmtB protein; ebh extracellular matrix-binding protein.

To analyze the adhesion and invasion capabilities of patient-derived *S. aureus* strains to osteoblast cells, MG63 and SaOS2 cells were seeded into 6-well plates and cultured to confluence. To assess the ability of *S. aureus* isolates to adhere to osteoblasts, confluent cell layers of SaOS2 or MG63 were incubated for 30 min with a bacterial suspension harvested during the stationary growth phase. Adhesion to the cells was compared to adherence to the plastic surface of the 6-well plates as a control. As illustrated in Figure 2A, isolates from patients 2, 4, 6, 9, 28, and 42 exhibited comparable adherence to cells and plastic, with isolate 42 showing approximately 30 times higher adherence to plastic or cells compared to the other conditions. Isolates from patients 36 and 45 demonstrated significantly elevated adherence to cells in contrast to the plastic surface of the 6-well plates. Interestingly, isolates from patient 36 and *S. aureus* ATCC^®^ 29213^TM^ exhibited differences in their ability to adhere to SaOS2 or MG63 cells, following that the different *S. aureus* isolates did not exhibit uniform adhesion properties.

Next, the invasion capabilities of the various *S. aureus* strains to the MG63 and SaOS2 cells were investigated. For this purpose, confluent layers of both cell lines were incubated with a bacterial suspension of *S. aureus* strains for 30 min, followed by an incubation in DMEM supplemented with 30 μg/mL gentamicin for selective elimination of extracellular bacteria while preserving intracellular ones. After one day, the cells were lysed, and the number of intracellular bacteria was determined by plating serial dilutions of the lysates on LB-agar plates and counting colony-forming units (CFUs). As depicted in Figure 2B, no differences in invasion capabilities were detected among the various *S. aureus* isolates.

Despite the high bacterial count observed for patient 36, 42, and 45 in the cells, isolates 2, 4, 6, 9, and 28 showed less prominent abilities to adhere to the osteoblasts. Isolates from patients 4, 28, 42, 45, and *S. aureus* ATCC^®^ 29213^TM^ showed significant invasion rates in osteoblasts as compared to control with a strong preference to SaOS2 cells, suggesting that the invasion properties of *S. aureus* strains are highly variable and depend on both the specific bacterial isolate and the osteoblast cell line.

Given the diverse adhesion and invasion properties of the patient-derived *S. aureus* isolates, the expression patterns of characteristic adhesion genes in these isolates were investigated. As shown in Figure 2C, the tested adhesion genes exept fnbB, bbp, can, ebp, and fib, could be detected in all isolates of *S. aureus*.

Among these, several adhesion-related genes including eno, fnbA, clfB, and ebpS were expressed in both adhesive and non-adhesive *S. aureus* isolates. Specifically, isolates from patients 36, 42, and 45, as well as *S. aureus* ATCC^®^ 29213^TM^ demonstrated higher expression levels of these genes regardless of their adhesive properties.

Interestingly, the gene clfA emerged as a potential key candidate, as it was overexpressed mainly in the strongly adhesive *S. aureus* isolates.

Simultaneously, the expression of corresponding adhesion proteins in *S. aureus* isolates were analyzed by mass spectrometry (Figure 2C). Proteome analysis of the different *S. aureus* strains revealed that the proteins, CflA, EbpS, and Ebh were found in all isolates, while the other proteins were only found in some but not all isolates, which does not mean that they do not occur in the proteome due to the method. Overall, the different gen and protein patterns are in line with previous findings, and they indicate that the adhesion and internalization process in *S. aureus* is not homogenously regulated nor conserved among the different bacterial strains.

### 2.3. Osteoblasts Successfully Survived S. aureus Infection, Independent of Bacterial Strain

**Figure 3:**
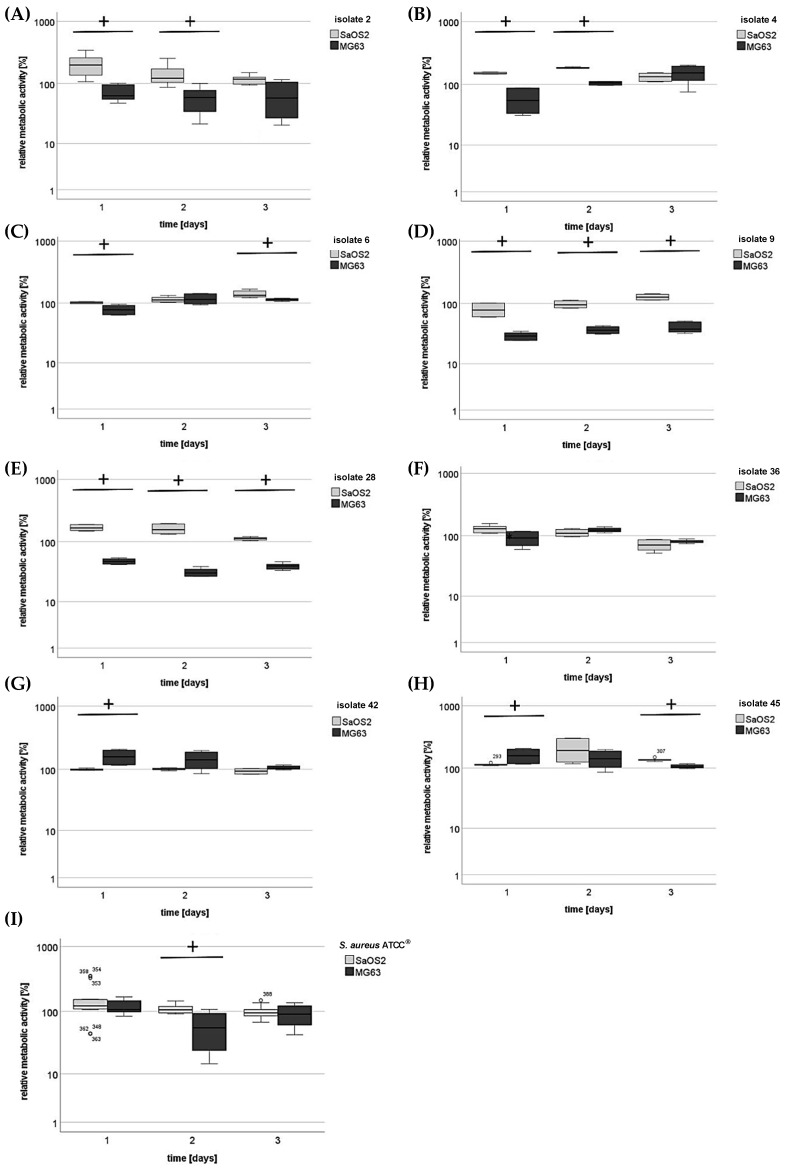
Cellular viability of osteoblasts after *S. aureus* infection. (**A**–**I**) Confluent layers of SaOS2 and MG63 osteoblasts were exposed to the different bacterial isolates (harvested during the stationary phase) for 30 min. Following incubation, non-adherent bacteria were removed by washing with PBS. Subsequently, DMEM supplemented with 10% FCS and 30 μg/mL gentamicin was applied to eliminate extracellular while preserving intracellular bacteria. After 1, 2, and 3 days metabolic activity was determined using MTT assay (+ *p* < 0.05 indicating significant difference between SaOS2 and MG63 cells; * *p* < 0.05 indicating a significant difference between single time points).

To assess whether the osteoblasts were affected by bacterial presence in the aforementioned experiments, MTT assays were conducted to measure metabolic activity as an indicator for cellular viability of the osteoblastic cells. In the course of 3 days, osteoblasts were infected with the patient-derived *S. aureus* isolates and analyzed by MTT assay after 1, 2, and 3 days (Figure 3A–I). Interestingly, these assays revealed, that cell viability of both SaOS2 and MG63 cells remained constant and unaffected irrespective the presence of any bacterial strain, suggesting a robust survival capability of osteoblasts in response to *S. aureus* infection.

### 2.4. Impact of S. aureus Infection on the Bioenergetic Profile of Osteoblasts

**Figure 4:**
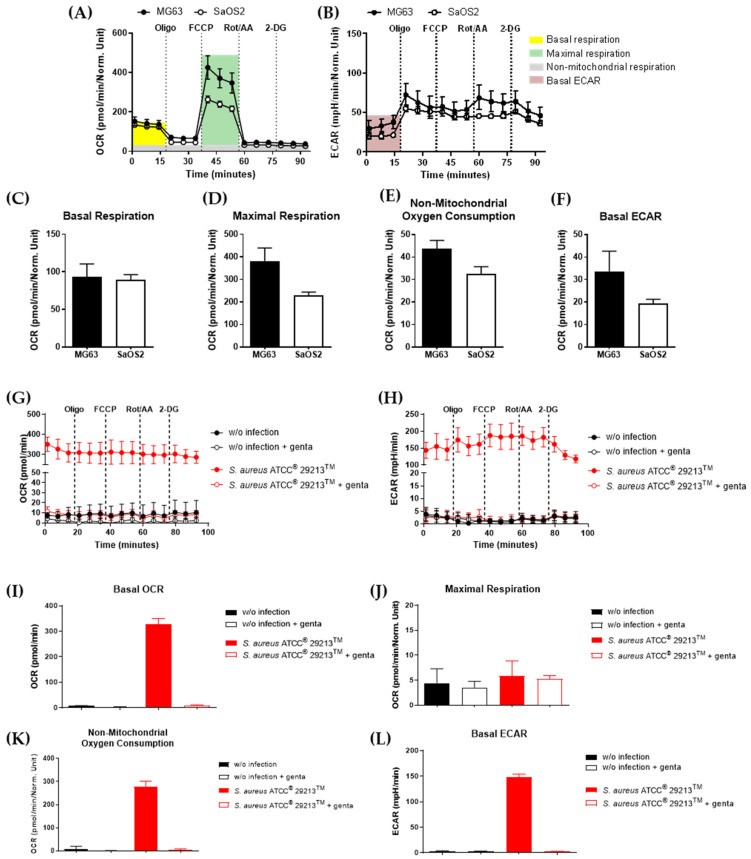
Osteoblasts and *S. aureus* differ in their bioenergetic profiles. (**A**) Oxygen consumption rate (OCR) and (**B**) extracellular acidification rate (ECAR) of MG63 and SaOS2 cells. The OCR was measured under basal and stressed conditions using Seahorse technology with a Mito Stress Test by sequential injections of oligomycin (Oligo), FCCP, rotenone/antimycin A (Rot/AA) 24 h after seeding. (**C**–**F**) A calculation of mitochondrial activity was performed as illustrated. Maximal respiration resulted from the OCR after the injection of FCCP minus the OCR after injection of oligomycin. Non-mitochondrial oxygen consumption rates were obtained by the OCR levels after Rot/AA injection. (**G**,**H**) Respiratory activity of *S. aureus* ATCC^®^ 29213^TM^ in osteoblast-free conditions after 24 h and (**I**–**L**) metabolic parameters calculated in accordance with (**A**), respectively. genta: gentamicin.

In addition to biofilm formation, *S. aureus* strains can invade osteoblasts and persist intracellularly. This provides a protected environment for the bacteria, allowing them to survive and potentially evade host immune responses and antibiotic treatments. Therefore, the metabolic response of osteoblasts under uninfected and infected conditions were studied separately using a Seahorse Extracellular Flux Analyzer. The resulting oxygen consumption rates (OCRs), were further distinguished into basal respiration, maximal respiratory capacity, and the non-mitochondrial OCR (Figure 4A). Simultaneously, the extracellular acidification rates (ECARs) were assessed, reflecting the glycolytic activity of the cells (Figure 4B).

First, OCR and ECAR of uninfected osteoblasts were compared after reaching confluence. These measurements revealed a slightly higher metabolic capacity of MG63 compared to SaOS2 osteoblasts, as indicated by the enhanced maximal respiration (Figure 4D), the non-mitochondrial oxygen consumption rate (Figure 4E), and the elevated basal ECAR levels (Figure 4F), underlining the results from the previous MTT assays.

To investigate the *S. aureus*-mediated respiration, the OCR and ECAR levels of *S. aureus* ATCC^®^ 29213^TM^ and patient-derived strains were assessed in a cell-free environment (Figure 4G,H; S1.Figure S1A–D). Therefore, *S. aureus* was incubated with and without gentamicin for 24 h. In the gentamicin supplemented *S. aureus* conditions, the bacteria were not able to survive, reflected by the unaltered OCR and ECAR levels compared to the *S. aureus*-free blanks. However, without gentamicin supplementation, *S. aureus* proliferation led to an increase in the OCR and ECAR levels, which was accompanied by a resistance against the injected electron transport chain inhibitors such as oligomycin, FCCP and rotenone/antimycin A, revealing a characteristic respiration profile for the bacterial strains compared to the osteoblast-mediated metabolic profile (Figure 4A,B).

**Figure 5:**
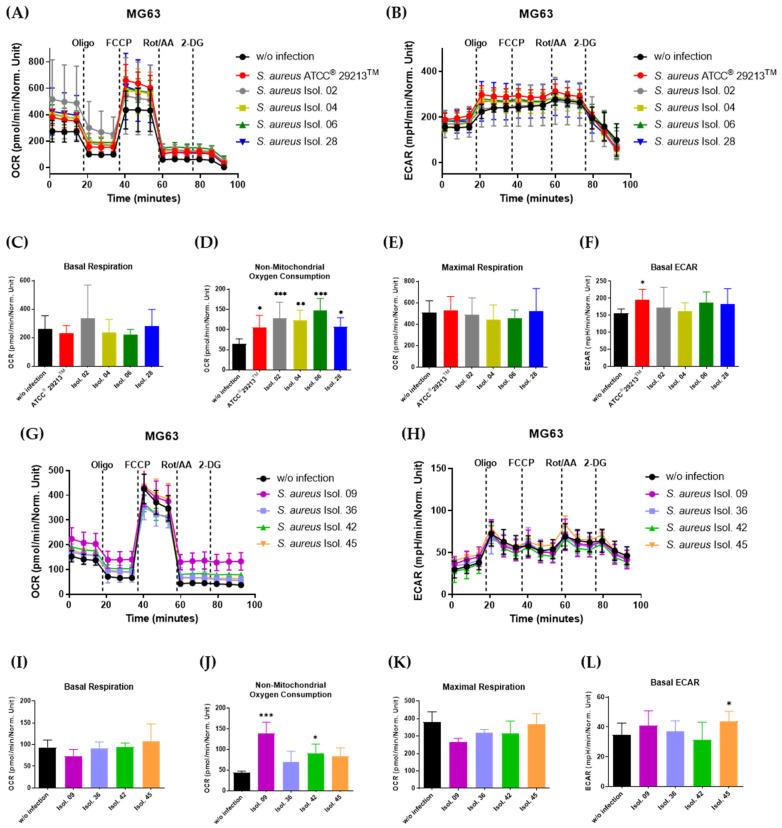
Mitochondrial respiration is altered in MG63 osteoblasts infected with *S. aureus* isolates. (**A**,**B**,**G**,**H**) MG63 cells were infected with isolates from patients 2, 4, 6, 9, 28, 36, 42, 45, and *S. aureus* ATCC^®^ 29213^TM^, followed by gentamycin supplementation in order to remove extracellular bacteria. After one day, respiration was analyzed by a Seahorse Mito Stress Test, followed by the quantification of the basal respiration (**C**,**I**), non-mitochondrial oxygen consumption (**D**,**J**), maximal respiration (**E**,**K**), and basal ECAR (**F**,**L**) from the obtained respiratory profiles. All results are representative of at least three independent experiments. Data are demonstrated as mean ± SD of at least six replicate samples. * *p* < 0.05, ** *p* < 0.01, and *** *p* < 0.001 compared to uninfected control, ANOVA, Bonferroni’s test.

Interestingly, infection of MG63 osteoblasts with any patient-derived *S. aureus* strains did not influence the basal respiration (Figure 5C,I) and the maximal respiratory capacity of the osteoblasts (Figure 5E,K), whereas the non-mitochondrial oxygen consumption of MG63 cells was significantly elevated by all implant-derived *S. aureus* strains, except isolate 36 and 45 (Figure 5D,J). On the other hand, *S. aureus* strains did not have a significant impact on the overall glycolytic activity, as confirmed by similar basal glycolytic activity among the uninfected and infected MG63 cells (Figure 5F,L).

**Figure 6:**
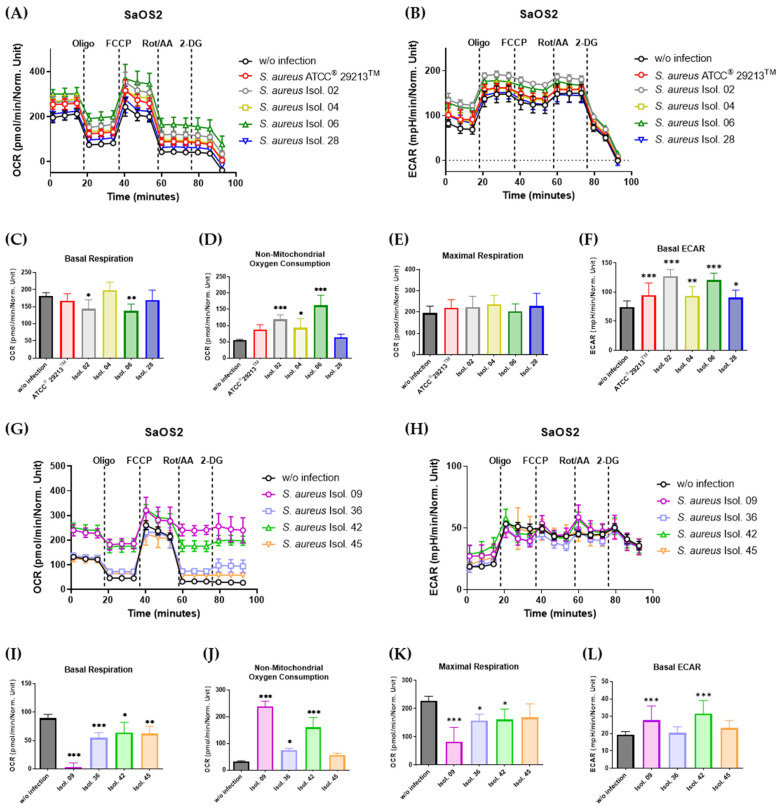
Mitochondrial respiration is altered in SaOS2 osteoblasts infected with *S. aureus* isolates. (**A**,**B**,**G**,**H**) SaOS2 cells were infected with isolates from patients 2, 4, 6, 9, 28, 36, 42, 45 and *S. aureus* ATCC^®^ 29213^TM^, followed by gentamycin supplementation in order to remove extracellular bacteria. After day one, respiration was analyzed by a Seahorse Mito Stress Test, followed by the quantification of the basal respiration (**C**,**I**), non-mitochondrial oxygen consumption (**D**,**J**), maximal respiration (**E**,**K**), and basal ECAR (**F**,**L**) from the respective respiratory profiles. All results are representative of at least three independent experiments. Data are demonstrated as mean ± SD of at least six replicate samples. * *p* < 0.05, ** *p* < 0.01, and *** *p* < 0.001 compared to uninfected control, ANOVA, Bonferroni’s test.

In contrast, SaOS2 cells responded more sensitively towards *S. aureus* infection compared to MG63 osteoblasts. As shown in Figure 6, isolates 6, 9, 36, 45 and *S. aureus* ATCC^®^ 29213^TM^ led to a significant decrease in the basal respiration, which was accompanied by an increase in the non-mitochondrial oxygen consumption rate in most of the infected SaOS2 osteoblasts (*S. aureus* ATCC^®^ 29213^TM^, Isol. 02, Isol. 06, Isol. 09, Isol. 36 and Isol. 42). Moreover, all conditions infected with *S. aureus* (except Isol. 36 and Isol. 45), revealed a significant increase in glycolytic activity compared to the uninfected control (Figure 6F,L).

These results clearly suggest that *S. aureus* is able to survive in both osteoblast cell lines, observed by the increase in non-mitochondrial OCR and the slight increase in the glycolytic activity. However, bacterial respiration occurred more pronounced in the SaOS2 osteoblastic cells, confirming that these cells were more susceptible to intracellular proliferation of *S. aureus* strains.

## 3. Discussion

*Staphylococcus aureus* is considered to be the predominant pathogen in implant-associated bone infections [37] and more pathogenic compared to other strains [14,38,39,40,41,42].

Aside from the formation of biofilms, which is a pivotal factor in implant infection pathogenesis [43], it is clear that *S. aureus* can be seen as an intracellular pathogen that can persist intracellularly in several cell types [44,45,46,47]. This leads to an escape from the immune system and antibiotic therapy and, therefore, plays an important role in the persistence and recurrence of infection [48].

Our results demonstrate that *S. aureus* is not only able to adhere to but also able to invade osteoblasts, which is linked to its virulence phenotype [44].

The ability to adhere to osteoblasts differed between the clinical isolates from implant-associated infections with isolate 42 showing the strongest adhesivity and with isolates 2, 4, 9, and 28 expressing weak adhesive capacities. Additionally, *S. aureus* ATCC^®^ 29213^TM^ exhibited significantly higher adhesion to SaOS2 cells compared to MG63 cells; however, the pronounced adhesion to SaOS2 cells was not conserved among the different implant-derived strains. In contrast, isolates 4, 6, 28, and especially isolates 36 and 45 adhered much more efficiently to MG63 cells than to SaOS2 cells.

Interestingly, the different adhesion properties were not correlated with the invasion of the isolates into the osteoblasts, which was demonstrated by high variations in invasiveness between the *S. aureus* strains and the diverse preference for SaOS2 or MG63 cells. Nonetheless, isolate 36 served as one of the most invasive *S. aureus* strains into SaOS2 as well as into MG63 osteoblasts among the tested isolates. These variations in the adhesive and invasive capabilities have been described for different human MRSA isolates [49] before. Our studies show that this also applies to other *S. aureus* isolates.

An extensive analysis of various pathogenic factors and adhesion genes was performed by PCR technique and mass spectrometry to elucidate the factors contributing to the differential invasion capabilities observed among the isolates.

These analyses showed that the mere presence or absence of a gene says nothing about the adhesiveness or invasiveness of an isolate. Thereby, the gene prevalence of fnbA, clfA, and clfB across all isolates underlined their significance in bacterial virulence, which is consistent with prior research [50]. Furthermore, the identification of the ebpS gene in all isolates pointed out its role in facilitating binding to host tissues, while the consistent presence of psm (phenol-soluble modulins) genes psmA or psmB highlighted their potential involvement in biofilm formation [51], contributing to antibiotic resistance. The detection of the eap gene in all isolates suggests its conserved role in modulating inflammatory responses and enhancing bacterial internalization [52,53]. However, due to heterogenous invasion properties of our different *S. aureus* isolates, the single presence of these genes cannot be seen as a predictive marker for cell infection.

In addition to the wide variation in expression levels of the clumping factor clfA, we found increased basal expressions mainly in the strongly adhesive *S. aureus* isolates 36, 42, 45, and *S. aureus* ATCC^®^ 29213^TM^, suggesting that only clfA may serve as a candidate gene associated with the adhesive phenotype. ClfA binds to fibrinogen and promotes bacterial attachment to plasma protein-coated biomaterials allowing the bacteria to colonize and form a biofilm [54].

Conversely, strains deficient in the expression of ClfA, ClfB, and the serine-aspartate repeat protein SdrD were shown to have no impact on osteoblast binding [55], questioning the role of these genes in osteoblast adhesion likewise.

Another important role was described for the cell surface integrin α5β1, binding Fn on the surface of human cells, allowing *S. aureus* to invade and internalize into endothelial cells and osteoblasts [56,57,58]. However, FnbA and FnbB were either not present in the genome of the isolates or their basal expression levels were similar among strong and less adhesive isolates in our study.

If so, it is independent of the basal expression level and might be regulated after infection of osteoblasts and interaction with *S. aureus*. This remains to be elucidated, requiring further investigation of its precise role in bacterial cell adhesion.

Interestingly, isolates with strong adhesion, such as isolate 42, exhibited invasion rates similar to those with weaker adhesion, indicating that adhesion strength may not necessarily dictate invasion capacity. Additionally, the ability of *S. aureus* isolates to survive intracellularly varied across both cell lines, with isolate 36 and, to some extent, *S. aureus* ATCC^®^ 29213^TM^ showing pronounced survival compared to other isolates. This aligns with the finding that osteoblasts may pose a greater challenge for bacterial invasion and intracellular persistence [59], and further reinforces recent studies showing strain-dependent elimination of *S. aureus* in various cell types including dendritic cells, macrophages, and epithelial cells [60,61,62], suggesting that the survival of *S. aureus* within host cells is strongly influenced by both bacterial characteristics and host cell factors.

In addition, the invasion of *S. aureus* isolates was particularly pronounced in SaOS2 cells, likely due to their mature osteoblast character, in contrast to MG63 cells expressing features of immature osteoblasts [63]. Since both osteoblastic cell lines produce a differently composed extracellular matrix [64], this can contribute to the different capabilities of *S. aureus* strains to adhere to the osteoblasts, especially collagen IV, which was demonstrated to be characteristically expressed by SaOS2 cells in contrast to MG63 cells, representing a potential adhesion factor for *S. aureus*. [64]. Furthermore, MG63 has an integrin subunit profile similar to human osteoblasts [65,66]. While this was not equivalent to SaOS2 cells, it implicated that the developmental state of the osteoblasts plays a certain role in the infection process, which may be underlined by the differences in metabolic turnover when comparing the extracellular flux of both MG63 and SaOS2 cells. However, all isolates were internalized by both cell lines and persisted for at least 24 h.

Interestingly, this internalization did not impact the viability of MG63 and SaOS2 cells, as evaluated by the MTT assays following infections with *S. aureus*. While metabolic activity of infected osteoblasts did not differ from that of non-infected cells, our results revealed a shift in the bioenergetic metabolism of the osteoblasts. This finding suggests that the decreased survival of *S. aureus* in SaOS2 cells may not be attributed to the death of infected osteoblasts. Instead, our findings identified differences in the metabolic adaptations of MG63 and SaOS2 cells towards infection.

In particular, we observed an increase in cellular respiration driven by an enhanced non-mitochondrial oxygen consumption rate in both osteoblastic cell lines and by most of the applied *S. aureus* strains, with strains 2, 6, 9, and 42 showing the most pronounced activities. Interestingly, in the SaOS2, but not in the MG63 osteoblasts, this was accompanied by a reduction in the basal OCR and an increase in the basal glycolytic activity. These metabolic shifts, on the one hand, may signify that *S. aureus* is able to survive and to co-exist in osteoblasts [67], but on the other hand, it indicates that distinct differentiation states of osteoblasts differ in their metabolic response towards *S. aureus* infection, which may have an impact for the clearance and eradication of the pathogens not only by the host cells themselves, but also during antibiotic treatment.

A key role for mitochondrial regulation is supported by recent findings in neutrophils, where mitochondrial ROS production during *S. aureus* infection was shown to be dependent on the ETC complexes [68,69]. In particular, the bactericidal activity of host cells was attenuated when mitochondrial ROS was inhibited by either the complex III inhibitor antimycin A or the antioxidant MitoTEMPO [68,69]. Moreover, in a mouse model for *S. aureus* prosthetic joint infection, it was shown that the *S. aureus* biofilm shifts monocytes towards OXPHOS over glycolysis [69,70]. Inhibition of oxidative metabolism in monocytes by the ATP synthase inhibitor oligomycin reprogrammed cellular metabolism in vivo, resulting in an increased production of pro-inflammatory cytokines and a significant reduction in *S. aureus* burden [69]. This suggests that specific inhibition of ETC complexes may promote the elimination of *S. aureus* in the host cells. This was confirmed when systemic antibiotics in combination with the reprogramming of monocyte metabolism, was able to effectively eradicate an established biofilm infection [69,70]. Together these results and our data imply that the effects of OXPHOS during *S. aureus* infection are cell- and context-dependent.

Interestingly, our results showed that mitochondrial respiration of the osteoblasts themselves was not necessarily altered, but non-mitochondrial respiration was increased in the cells, which was not due to an increased rate of glycolysis, applying in particular for the MG63 osteoblasts, where no differences in ECAR of infected cells compared to the untreated controls was observed, suggesting that oxygen is consumed in other ways in the host cells. This may be due to alternative energy production pathways by the different *S. aureus* strains, whose energy supply is not based on mitochondrial OXPHOS activity but is instead provided by distinct metabolic pathways such as lactate consumption.

The production and fermentation of lactate by *S. aureus*, belongs to the most important survival strategies of *S. aureus* in the infection process, especially during biofilm formation, when aerobic or anaerobic conditions can shape the environment of *S. aureus.* In these conditions, lactate alters the pH and local environment, allowing the pathogens to invade the host cells [71]. This was observed in biofilm-associated prosthetic joint infections, resulting in an increase in histone deacetylase (HDAC) activity and elevated anti-inflammatory cytokine IL-10 production [71,72]. On the other hand, it was demonstrated that *S. aureus* shares glycolytic pathways in the host inflammatory machinery. This was essential to support the growth and pathogenicity of *S. aureus* within the host cells such as macrophages [73,74].

These metabolic adaptations following infection were observed especially in immune cells, including macrophages, undergoing a metabolic shift with increased glycolysis and altered mitochondrial function, leading to pro-inflammatory immune responses. These changes were promoted by HIF-1α activation, the accumulation of TCA cycle metabolites, IL-1β, and IL-18 release. These immune responses towards *S. aureus* infection and biofilm formation have been recently reviewed in detail by Arumugam et al. [69] and Lung et al. [75]. However, similar mechanistic observations were made in keratinocytes infected with *S. aureus*, when glycolysis of *S. aureus* was necessary to infect these keratinocytes but also to meet the demands of the infection [76,77,78]. Most importantly, *S. aureus* internalization into the osteoblast-like MG63 cells was likewise associated with an increase in IL-1β but also with caspase-1 production and inflammasome activation in the osteoblastic cells [79]. Such pro-inflammatory characteristics are closely linked to metabolic reprogramming, particularly involving the TCA cycle and mitochondrial function. This may play an important role for pathogen clearance and for host defenses against *S. aureus* infections but also represents an indicator for the less pronounced survival of *S. aureus* in the SaOS2 cells compared to MG63 osteoblasts, since a metabolic shift to glycolysis was much more pronounced in the SaOS2 cells. Moreover, during such an acute host response towards infection, the IL-1β release was found to initiate the recruitment and activation of phagocytes to clear *S. aureus*.

Another important metabolite supporting the survival of *S. aureus* in host cells, is the TCA cycle intermediate fumarate, which was shown to be strongly metabolized by *S. aureus* to malate. The upregulation of fumarate hydratase (fumC) was identified to promote this increased metabolic turnover, leading to reduced levels of fumarate in the infected tissue, and by this to protect against trained immune reactions [76,80]. The extent to which these aspects affected OXPHOS activity is controversial. However, it was observed that the ability of *S. aureus* to switch between metabolic pathways has a key role for survival and promotion of chronic infection; therefore, different TCA-cycle associated genes are currently under investigation for targeted therapy [76,78,81].

In contrast, the metabolic shift induced by *S. aureus* infection in osteoblasts has been shown to disrupt mitochondrial function, and to impair mitochondrial respiration, causing osteomyelitis among others [82]. In response to mitochondrial dysfunction, osteoblasts increased their reliance on glycolysis to meet their energy demands [83]. This metabolic shift is often less efficient and can lead to the release of inflammatory cytokines [83,84,85], accumulation of lactate, and a more acidic intracellular environment [83,86], which not only supports bacterial survival and growth but also creates a hostile environment for the host cells along with implications for osteoblast formation [87]. Recently it was discovered that this metabolic shift induced by *S. aureus* is dominated by itaconate production and serves as a signaling factor for the immunometabolic response in osteoblasts, and, importantly, is associated with enhanced biofilm formation [88].

Moreover, this effect was often accompanied with increased mitochondrial membrane potential and elevated production of reactive oxygen species (ROS), causing oxidative stress and damage to cellular components, which was demonstrated to be an important initiation factor not only for osteoclast formation [89,90,91,92] but also for the activation of apoptosis or necroptosis in the osteoblasts [78,93]. This is particularly problematic for bone healing and regeneration, and it can result in the compromised ability to produce bone matrix, further leading to osteomyelitis and presenting challenges in the treatment of bone infections.

Our observations also reflect the ongoing clinical challenges in the treatment of osteomyelitis due to the ability of *S. aureus* to persist intracellularly and to adapt phenotypically, particularly through small-colony variants (SCVs). SCVs are quasidormant phenotypes characterized by slow growth, altered metabolism, and reduced virulence, rendering them less susceptible to antibiotics and immune clearance [94,95]. These variants persist in intracellular reservoirs and biofilms, and their development can even be promoted by antibiotics such as trimethoprim–sulfamethoxazole (TMP-SMX) and aminoglycosides [96,97]. Clinically, SCVs are prevalent, particularly in prosthetic joint infections, where they dominate over wild-type strains [34,97].

Despite their central role in infection persistence, treatment options targeting intracellular *S. aureus* and SCVs remain limited. Most available antibiotics are ineffective against intracellular pathogens due to poor cell penetration or diminished activity within host cells [95,98]. Promising candidates like teicoplanin, quinupristin/dalfopristin, and ofloxacin, as well as intracellular-active agents such as rifampicin, linezolid, and oritavancin, require further evaluation in bone-relevant models [95]. Additionally, new antibiotics like doxycycline and omadacycline have not been tested against *S. aureus* in intracellular osteomyelitis models [95].

## 4. Materials and Methods

### 4.1. Bacterial Strains

*Staphylococcus aureus* ATCC^®^ 29213^TM^, was purchased from the American Type Culture Collection (ATCC^®^ 29213^TM^, Manassas, VA, USA).

Clinical isolates were obtained during revision surgery of infected prosthesis. Approval was granted by the ethics committee of our institution (Az: 116/17), and informed consent was obtained in all cases. All microbiological samples were completely de-identified and stripped of all patient-identifying information. After the prostheses were removed, they were buried under sterile conditions in the laboratory and existing biofilms were removed with ultrasound (see below). Samples were collected from 50 patients; of these, 12 valid *S. aureus* isolates were selected for subsequent experiments and designated as patient 2, 4, 6, 9, 28, 36, 42, and 45.

### 4.2. Detachment of Bacteria by Sonication According to Esteban et al., 2008 [99]

Bacteria seeded devices were placed and hermetically closed in either 20 by 40 cm sterile plastic bags or glass boxes with 50 ml of sterile phosphate-buffered saline (PBS, pH 6.8). Then, samples were sonicated using BactoSonic ultrasonic bath (Bandelin, Berlin, Germany) for 2 min. Then, the sonicate was transferred to 50 ml Falcon tubes and centrifuged at 3000× *g* for 20 min (Heraeus Labofuge 400 R, Hereaus, Hanau, Germany), and the supernatant was discharged. The sediment was resuspended in 5 ml PBS and plated out on Columbia Agar with 5% Sheep Blood or LB Agar in various dilution stages in order to determine the colony forming units.

### 4.3. Characterization of the Staphylococci Isolates

Based on their morphology, growth appearance and agility, isolates suspected to be *Staphylococcus* were selected for detailed characterization. Therefore, the Gram-stain, growth behavior, enzymatic properties such as collagenase and oxidase tests, and 16S rRNA were analyzed.

Collagenase test

To differentiate *S. aureus* from coagulase-negative staphylococci, the Collagenase Test Kit (VWR Chemicals, Darmstadt, Germany) was used. The lyophilisated reagent was reconstituted by adding 10 mL of sterile water pre-warmed to 37 °C. The solution was then aliquoted into tubes, each tube containing 0.3 mL of the rehydrated medium.

For analysis, a loopful of bacterial isolate colony was inoculated directly into each tube. The tubes were incubated at 37 °C under static conditions and coagulation was monitored at multiple time points: 30 min, 1 h, 4 h, 6 h, and 24 h. The solidification of the medium indicated the presence of *S. aureus* or other coagulase-positive staphylococci.

Oxidase test

To determine bacterial strains based on their cytochrome c oxidase activity, the Oxidase Reagent Kit (BioMérieux, Marcy l’Étoile, France) was employed. Cytochrome c oxidase catalyzes the oxidation of the substrate N,N,N′,N′-tetramethyl-p-phenylenediamine dihydrochloride. This reaction produces a distinct color change from colorless to purple within 10 to 30 s, a characteristic feature of Neisseria and the majority of Pseudomonas species.

API^®^ Staph. Test

For the identification and differentiation of Staphylococcus species, the API Staph test (BioMérieux, Marcy l’Étoile, France) was performed. Homogeneous bacterial suspensions were prepared from 18 to 24 h-old cultures of the isolates and used to inoculate the API Staph medium. Following inoculation, the microtubes on the API Staph test strip were filled with the prepared medium in accordance with the manufacturer’s instructions and incubated at 37 °C for 18 to 24 h.

After incubation, specific reagents were added to the microtubes as required, and the identification of bacterial species was performed based on color changes observed in the microtubes, interpreted following the manufacturer’s instructions.

16S rRNA sequencing

16S rRNA sequencing was used to analyze and identify Staphylococcus species based on differences in their 16S ribosomal RNA (rRNA) genes. First, DNA isolation was conducted following the protocol described below. Then, the 16S rRNA gene was amplified via polymerase chain reaction (PCR) utilizing specific primers (27 Forward: 5′-AGA GTT TGA TCM TGG CTC AG-3′; 1492 Reverse: 5′-GGT TAC CTT GTT ACG ACT T-3′) [100]. PCR product purity was assessed by electrophoresis through 1% agarose gels (Carl Roth GmbH, Karlsruhe, Germany) stained with ethidium bromide (ETHBR). Subsequently, 5 μL of PCR products underwent purification with 1 μL of Exonuclease I (Exo I) and 2 μL of Shrimp Alkaline Phosphatase (rSAP) (New England Biolabs, Ipswich, MA, USA) per DNA product. The samples were incubated in a PCR device (Eppendorf AG, Hamburg, Germany) at 37 °C for 15 min followed by 80 °C for 15 min. DNA quantification was performed, and a total volume of 15 μL was collected for commercial analysis (Microsynth SEQLAB, Göttingen, Germany). The collection included 3 μL of the 27 forward primer, PCR product, and nuclease-free water, following the calculation formula provided in the company’s instructions. Upon receiving results from the company, isolate sequences were aligned with known sequences of *S. aureus* using BLAST: Basic Local Alignment Search Tool (nih.gov) [101].

### 4.4. Culture of Staphylococcus aureus

The bacteria were incubated overnight at 37 °C on Columbia blood agar plates (Merck KGaA, Darmstadt, Germany), and formed colonies were transferred to standard LB medium (Luria/Miller, Carl Roth GmbH, Karlsruhe, Germany) for subsequent experiments [102].

### 4.5. Biofilm Formation

Biofilm formation was examined for all isolates in two different media: LB-medium and LB-medium supplemented with 0.5% glucose and 3% NaCl. The *S. aureus* isolates were cultured in the 96-well plates for one day. Then, bacteria were stained using crystal violet (Sigma, St Louis, MO, USA), followed by a washing step with PBS, elution with 100% ethanol and quantification of the grown biofilm by OD570.

### 4.6. Bacterial DNA Isolation and PCR

The chromosomal DNA utilized as an amplification template was extracted from bacterial cultures using the innuPREP Bacteria DNA kit (Analytik Jena AG, Berlin, Germany) following the manufacturer’s instructions. PCR was performed as previously described in [103,104] with the primer sets bbp (encoding bone sialoprotein binding protein), cna (collagen binding protein), eno (enolase), fnbB (fibronectin binding protein B), fib (fibronectin binding protein), clfA, clfB (clumping factors A and B) [103], fnbA (fibronectin binding protein A), ebpS (elastin binding protein) [105], psmA, psmB (phenol-soluble modulins A and B) [106], arcB (ornithine carbamoyltransferase) [107], eap (extracellular adherence protein) [108], sdrD (SD-repeat containing protein D) [109], fmtB (methicillin resistance determinant FmtB protein) [110], ebh (extracellular matrix-binding protein) [111], and 16S as housekeeping gene with minor modifications [112]. All primers were purchased from Microsynth SeqLab (Goettingen, Germany).

The PCR reactions were performed in a total volume of 20 μL, consisting of 0.8 μL of each primer, 1 μL of extracted DNA, 0.1 μL of MyTaq DNA polymerase (BIOLINE GmbH, Luckenwalde, Germany), 4 μL of 5× MyTaq Red Reaction Buffer (BIOLINE), and 14.1 μL of nuclease-free water (BIOLINE). The thermal cycling conditions included an initial denaturation at 94 °C for 1 min, followed by 40 amplification cycles with denaturation at 94 °C for 30 s, annealing at 50–55 °C for 30 s, and extension at 72 °C for 30 s.

After amplification, 5 μL of the resulting amplicon was analyzed alongside a 1 kb DNA ladder (New England Biolabs, Ipswich, MA, USA) as a size marker. Electrophoresis was conducted on 1% agarose gels containing ethidium bromide (EtBr) at 70 V for 90 min. Gels were subsequently visualized under UV illumination and documented photographically.

### 4.7. Bacterial RNA Isolation and Real-Time RT-PCR/qPCR Analysis

Bacterial RNA was extracted using the peqGOLD Total RNA Kit (PeqLab, Erlangen, Germany) following the manufacturer’s instructions. The resulting RNA concentrations were measured using spectrophotometry.

cDNA synthesis for reverse transcription PCR (RT-PCR) was performed with the iScript cDNA Synthesis Kit (Bio-Rad, Hercules, CA, USA) in line with the manufacturer’s protocol. The 20 μL reaction mixture contained 5 μL of RNA template, 4 μL of 5× iScript reaction mix, 1 μL of iScript reverse transcriptase, and 10 μL of nuclease-free water. Reactions were conducted in a PCR thermocycler using the following protocol: 5 min at 25 °C, 30 min at 42 °C, and 5 min at 85 °C to generate cDNA.

Real-time PCR was performed on a Realplex2 Mastercycler (Eppendorf AG, Hamburg, Germany) using the SsoAdvanced Universal SYBR^®^ Green Supermix (Bio-Rad Laboratories, Hercules, CA, USA). Each 20 μL reaction mixture consisted of 1 μL of cDNA template, 2 μL of 10× forward and reverse primers, 7 μL of nuclease-free water, and 10 μL of SYBR^®^ Green Supermix. The PCR protocol comprised an initial denaturation at 95 °C for 30 s, followed by 40 cycles of denaturation at 95 °C for 15 s, annealing at 55 °C for 35 s, and extension at 95 °C for 15 s. Melting curve analysis was performed by incrementally raising the temperature from 65 °C to 95 °C at 1 °C per minute over 30 min.

Gene expression levels were assessed using the delta-delta Ct method, with threshold cycle data obtained from the melting curve analysis.

### 4.8. Cultivation of Osteoblasts

Human musculoskeletal osteoblast-like cell lines MG63 (ATCC^®^ CRL-1427™) and SaOS2 (ATCC^®^ HTB85™) were maintained in DMEM (Gibco, Karlsruhe, Germany) supplemented with 10% fetal calf serum (Biochrom, Berlin, Germany) and 1% penicillin/streptomycin (10,000 U/mL; PAA Laboratories, Pasching, Austria). Cells were cultured at 37 °C in a humidified Heracell™ 150i incubator with 5% CO_2_ (Heraeus, Hanau, Germany) in 75 cm^2^ flasks (SARSTEDT, Nümbrecht, Germany). Once sub-confluence was reached, cells were detached using 1 mL trypsin-EDTA and washed with phosphate-buffered saline (PBS) [113].

For experimental procedures, cells were seeded into 6-well plates and allowed to grow to confluence. Transmitted light microscopy was used to monitor cell growth and confluence.

### 4.9. S. aureus Adhesion Assay

*S. aureus* was cultured in an LB-Medium and harvested during the stationary or logarithmic phase via centrifugation. The resulting pellet was washed twice with PBS. Next, the pellet was resuspended in DMEM supplemented with 10% FCS, and the bacterial concentration was calibrated and adjusted based on optical density at 600 nm.

In between, osteoblasts were cultured in DMEM containing 10% FCS and 1% penicillin/streptomycin (10,000 U/mL) until sub-confluence. Osteoblasts were then detached using 0.05% Trypsin (Gibco Thermo Fisher Scientific GmbH-Dreieich, Germany), seeded at a density of 0.3 × 10^6^ cells into 6-well plates (SARSTEDT, Nümbrecht, Germany), and allowed to grow overnight to reach a confluent layer. Subsequently, the culture medium was replaced with the *S. aureus* suspension, and the SaOS2 and MG63 osteoblasts were infected for incubation time points as indicated at 37 °C in a 5% CO_2_ humidified atmosphere.

After incubation, the supernatant containing the bacteria was discarded, and cells were washed five times (4 mL per wash) with 1× PBS to remove unattached bacteria. Then, the cells were lysed with 0.2% Triton X-100 for 20 min and thoroughly mixed to achieve complete lysis. The resulting lysates were diluted tenfold (1:10, 1:100, 1:1000, 1:10,000) in 1× PBS and plated onto LB-agar plates. Following 24 h of incubation at 37 °C, the number of bacterial colonies was counted, and the total colony-forming units (CFU) were determined. Inter and intra assay precision were 53 and 63, respectively.

### 4.10. Invasion and Suvival Assay

For the invasion assay, DMEM supplemented with 10% FCS and 30 μg/mL gentamicin was added to the cells for selective elimination of extracellular bacteria while preserving intracellular ones, as described by Drevets et al. [114]. Then, cells were cultured one day before being lysed. Inter and intra assay precision were 39 and 47, respectively.

### 4.11. MTT Assay

The MTT assay was performed to evaluate the metabolic activity of MG63 and SaOS2 osteoblast cell lines, both with and without infection. Following incubation for 1, 2, and 3 days, the culture medium was aspirated, and 1 mL of MTT reagent (Thiazolyl Blue Tetrazolium Bromide, Sigma-Aldrich, St. Louis MO, USA) at a concentration of 5 mg/mL in PBS was added to the cells. Subsequently, the osteoblasts were incubated at 37 °C for a minimum of 4 h. Following the incubation period, 1 mL of Dimethyl sulfoxide (DMSO; Uvasol^®^, Darmstadt, Germany) was applied into each well containing the osteoblasts. The resulting purple solution was then diluted with DMSO and the absorbance was measured spectrophotometrically at OD550.

### 4.12. Seahorse Measurements

MG63 and SaOS2 osteoblastic cells were seeded in XFe96-well plates (Seahorse Bioscience, Billerica, MA, USA) at 10,000 cells per well and cultured overnight. After adhesion, cells were infected with *S. aureus* strains for 30 min, washed with PBS, and incubated in a medium with gentamicin for 24 h. On the day of measurement, cells were incubated in Seahorse XF assay medium (4.5 g/L glucose, 2 mM glutamine, 1 mM pyruvate, pH 7.35) for 60 min at 37 °C in a non-CO_2_ incubator.

Real-time oxygen consumption rate (OCR) and extracellular acidification rate (ECAR) measurements were then performed using the XFe96 Extracellular Flux Analyzer (Agilent Technologies, Santa Clara, CA, USA). Baseline respiration and glycolysis were assessed by three initial measurements, followed by sequential injections of metabolic modulators with a final concentration of 3 µM oligomycin, 0.5 µM FCCP, 100 nM/1 µM rotenone/antimycin A, and 50 mM 2-deoxyglucose.

Key parameters were calculated from averaged data of three measurement points and analyzed using Seahorse Wave software 2.6.1 (Agilent Technologies, Santa Clara, CA, USA).

### 4.13. Protein Identification by Mass Spectrometry

*S. aureus* isolates were cultured to stationary growth phase as described previously. For protein identification, mass spectrometry was utilized. Cell lysates were prepared by dissolving bacteria pellets in a lysis buffer containing 8 M urea and 0.1 M ammonium bicarbonate (Sigma, Saint Quentin Fallavier, France). After vortexing and ultrasonication, samples were centrifuged, and protein concentration was measured using the Bradford assay with bovine serum albumin (BSA) as a reference curve. Aliquots of 100 µg of protein for each was used. Then, 8 M Urea was added to give a final volume of 50 µL. If necessary, pH of the samples was adapted with 0.1 M HCl or NaOH to 7–9. 1.25 µL of a 0.2 M TCEP solution in 0.1 M NH_4_HCO_3_ was added to each sample, and samples were incubated for 1 h in a shaker at 37 °C and 1000 rpm. After cooling of the samples to 25 °C, 1.25 µL of a 0.4 M iodoacetamide solution in bidest water was added to the samples. Subsequently, samples were incubated in the dark at 25 °C and 500 rpm. Following, 1.25 µL of a 0.5 M N-acetyl-cystein solution in 0.1 M NH_4_HCO_3_ was added to each sample, and samples were incubated for 10 min at 25 °C and 500 rpm. If necessary, pH was adjusted to 8–9. Then, the urea concentration was decreased to 6 M urea by dilution with 0.1 M NH_4_HCO_3_ and 2.5 µL of a 0.2 µg/µL LysC solution in 0.1 M NH_4_HCO_3_ were added. Samples were then incubated at 37 °C for 3 h. Subsequently samples were further diluted using 0.1 M NH_4_HCO_3_ to give a final urea concentration of 1.6 M. Following, 4 µL of a 0.5 µg/µL solution of Sequencing Grade Modified Trypsin (Serva, Heidelberg, Germany) in 0.1 M NH_4_HCO_3_ were added. Samples were incubated at 37 °C overnight. TFA was added to a final concentration of approximately 1% to give a pH below 2. Samples were centrifuged at 14,000 rpm.

Peptides were desalted and concentrated using Chromabond C18WP spin columns (Macherey-Nagel, Düren, Germany, Part No. 730522). Finally, peptides were dissolved in 25 µL of water with 5% acetonitrile and 0.1% formic acid. Peptide concentration was measured using a Nanodrop (Thermo Scientific, Waltham, MA, USA) and samples were diluted accordingly to 100 ng of peptides per µL.

Mass spectrometric analysis of the samples was performed using a timsTOF Pro mass spectrometer (Bruker Daltonic, Billerica, MA, USA). A nanoElute HPLC system (Bruker Daltonics), equipped with an Aurora column (25 cm × 75 µm) C18 RP column filled with 1.7 µm beads (IonOpticks, Fitzroy, Australia) was connected online to the mass spectrometer. A portion of approximately 200 ng of peptides corresponding to 2 µL was injected directly on the separation column. Sample loading was performed at a constant pressure of 800 bar. Separation of the tryptic peptides was achieved at a 60 °C column temperature with the following gradient of water/0.1% formic acid (solvent A) and acetonitrile/0.1% formic acid (solvent B) at a flow rate of 400 nL/min: linear increase from 2%B to 17%B within 36 min, followed by a linear gradient to 25% solvent B within a min, and a linear increase to 37% solvent B in an additional 6 min. Finally, solvent B was increased to 95% within 10 min and held for additional 10 min. The built–in “PASEF long-gradient” method developed by Bruker Daltonics was used for mass spectrometric measurement. Protein identification and quantification was performed using “Proteome Discoverer 2.4” (ThermoFisher Scientific, Bremen, Germany) with Uniprot databases.

### 4.14. Statistical Analysis

Statistical analysis was conducted using IBM SPSS Statistics 27. The data were derived from at least three independent experiments and assessed for normal distribution with the Kolmogorov–Smirnov test. Results were compared using appropriate statistical tests, including Student’s *t*-test, Mann–Whitney U test, Kruskal–Wallis test, followed by ANOVA, as applicable. A *p*-value of <0.05 was considered statistically significant.

The results from both the adhesion and invasion assays were presented as a boxplot of adherent or internalized bacteria relative to the initially inoculated bacteria or as mean ± standard variation for seahorse experiments.

## 5. Conclusions

In summary, our findings point out that the presence of specific adhesion genes or their proteins cannot serve as reliable predictive markers for bacterial adhesion in *S. aureus* infections. Importantly, we unveiled a key role of non-mitochondrial respiration in osteoblasts upon *S. aureus* infection, indicating that intracellular bacteria are still metabolically active, which may have implications for the initiation of pro-inflammatory signals, changes in the cell homeostasis and cell death, creating the basis for cellular pathological changes.

Overall, these findings contribute to a deeper understanding of the mechanisms underlying *S. aureus* osteoblast interactions and offer valuable insights into the infection dynamics of clinically relevant *S. aureus* strains, which are not uniformly conserved compared to commercially available *S. aureus*. Consequently, the present study highlights the need to elucidate both the specific virulence factors and host cell responses driving the observed variability in bacterial behavior, as well as to develop more effective strategies to tackle *S. aureus* infections.

## Data Availability

Original research data are available on reasonable request.

## Supplementary Materials

The following supporting information can be downloaded at: https://www.mdpi.com/article/10.3390/antibiotics14020119/s1, Figure S1: (A,C) Respiratory activities of *S. aureus* strains in osteoblast-free conditions after 24 h and (B,D) metabolic parameters calculated in accordance to (A,C) respectively. genta: gentamicin.
