# Supplementary material for: Implant-Derived S. aureus Isolates Drive Strain-Specific Invasion Dynamics and Bioenergetic Alterations in Osteoblasts
Source: Antibiotics (Basel). 2025 Jan 23;14(2):119. doi: 10.3390/antibiotics14020119 (PMC11852183; doi:10.3390/antibiotics14020119)
Supplement: Supplementary file 1 [file antibiotics-14-00119-s001.zip › antibiotics-3389623-supplementary.pdf]

Supplement S1: Respiratory profiles of implant-derived *S. aureus* isolates

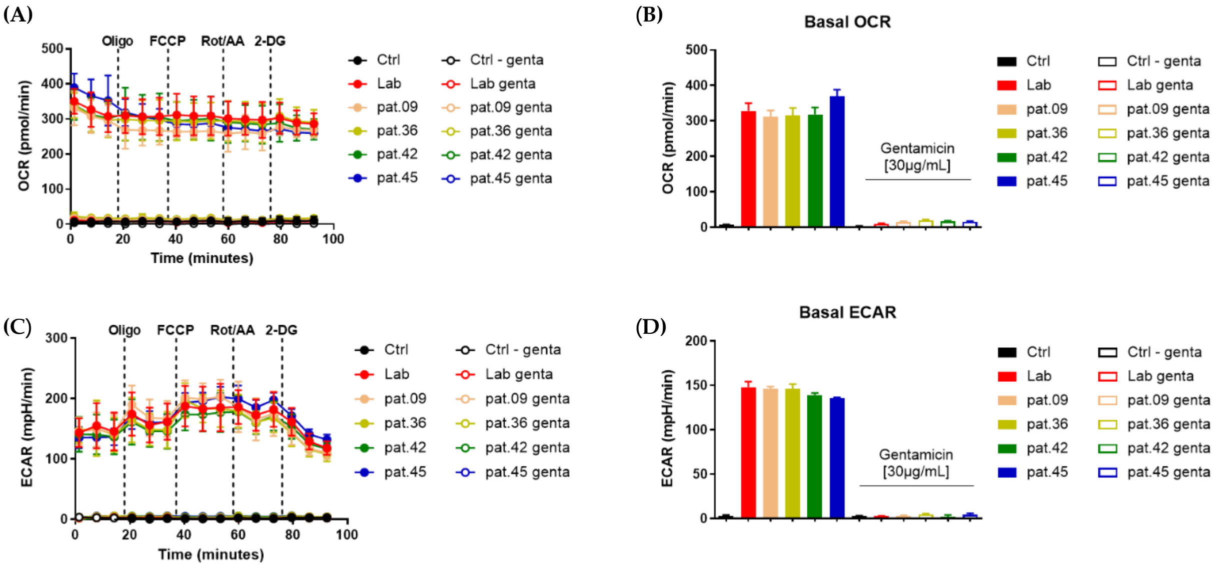

Figure S1: (A+C) Respiratory activities of *S. aureus* strains in osteoblast-free conditions after 24 h and **(B +D)** metabolic parameters calculated in accordance to (A+C) respectively. genta: gentamicin
